# Heterogeneity of epidermal growth factor receptor mutations in lung adenocarcinoma harboring anaplastic lymphoma kinase rearrangements: A case report

**DOI:** 10.3892/ol.2014.2468

**Published:** 2014-08-21

**Authors:** QIONG SUN, JIAN-YU WU, SHUN-CHANG JIAO

**Affiliations:** Department of Oncology, Chinese People’s Liberation Army General Hospital, Beijing 100853, P.R. China

**Keywords:** anaplastic lymphoma kinase rearrangement, crizotinib, epidermal growth factor receptor mutation, erlotinib, non-small cell lung cancer

## Abstract

Lung cancer is a heterogeneous and complex disease that remains the leading cause of cancer-related mortality worldwide. The identification of epidermal growth factor receptor (EGFR) mutation and anaplastic lymphoma kinase (ALK) rearrangements has changed the treatment of non-small cell lung cancer, creating a personalized treatment era that is based on the appropriate molecular selection of patients. In spite of the efficacy of tyrosine kinase inhibitors (TKIs), acquired resistance remains inevitable due to various mechanisms. The present study reports the case of a 30-year-old patient with stage IV lung adenocarcinoma initially harboring an EGFR mutation. However, following disease progression and a series of treatments, the wild-type EGFR gene was observed and the ALK rearrangements were revealed. Erlotinib administration resulted in a good response in the patient initially, but crizotinib did not. This indicated an association between the secondary mutations in kinases and the drug resistance to TKIs. This case should also highlight the clinical significance of repeat biopsies for the subsequent therapeutic choices at the onset of clinical progression.

## Introduction

As a heterogeneous and complex disease, lung cancer is a challenge to treat (1). The change to personalized treatment based on appropriate patient selection has been advanced by the identification of epidermal growth factor receptor (EGFR) mutations and anaplastic lymphoma kinase (ALK) rearrangements in non-small cell lung cancer (NSCLC) (2). The incidence of echinoderm microtubule-associated protein-like 4 (EML4)-ALK fusion and EGFR mutation in patients with NSCLC is 7 and 15%, respectively. Previous studies have indicated that functional ALK rearrangement is mutually exclusive with other known activating mutations, such as the EGFR and KRAS mutations (3). However, the coexistence of EML4-ALK fusions and EGFR mutations has been reported continuously in patient case studies (4–8). The possibility of coexisting ALK fusions and EGFR or KRAS mutations would have a profound impact on the choice of therapy and would affect clinical laboratory workflow (3). Secondary mutations in kinases may be a common mechanism of drug resistance to kinase inhibitors (TKIs) (9). The aim of the present study was to investigate the association between secondary mutations and acquired resistance to TKIs.

## Case report

**Figure 1:**
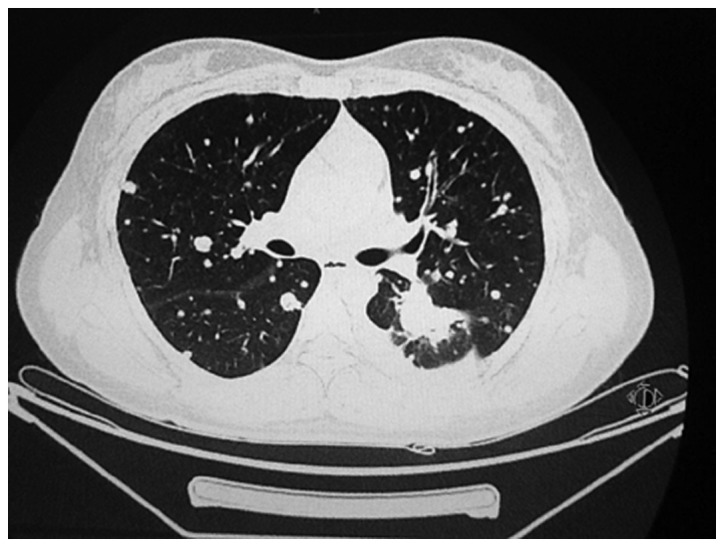
Initial chest computed tomography scan revealing a mass of 31×26 mm in size in the left lower lobe and metastases in each lung.

**Figure 2:**
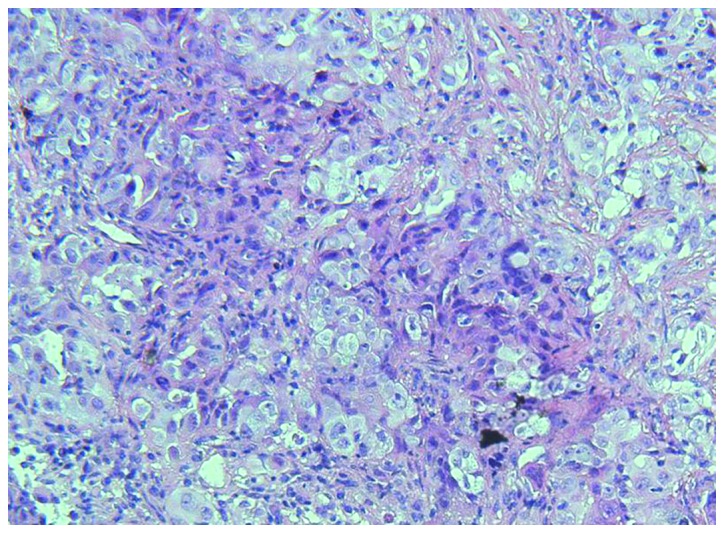
Histology of the primary tumor showing a poorly-differentiated adenocarcinoma (hematoxylin and eosin staining; magnification, ×200).

A 30-year-old female who had never smoked was referred to the Chinese People’s Liberation Army General Hospital (Beijing, China) for a persistent dry cough in July 2010. A chest computed tomography (CT) scan revealed a mass of 31×26 mm in size in the left lower lobe, and enlarged hilar lymph nodes and metastases in each lung (Fig. 1). A trans-bronchial lung biopsy (TBLB) was performed. The pathological diagnosis of the TBLB specimen was of a poorly-differentiated adenocarcinoma (Fig. 2). The laboratory findings were within the normal ranges, with the exception of a carcinoembryonic antigen level of 45.34 μg/l (normal range, 0–5.0 μg/l) in the serum. According to the clinical and histological findings, the patient was diagnosed with stage IV pulmonary adenocarcinoma (cT4N1M1a).

**Figure 3:**
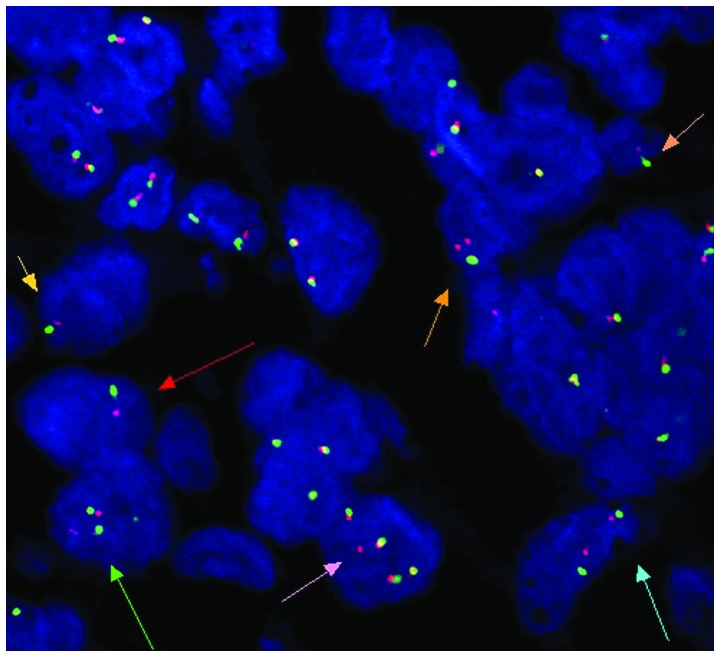
Signals for 2p23 proximal and distal probes for the ALK gene are indicated by green and red dots, respectively. The superimposed signals of 2p23 proximal and distal probes are indicated by yellow dots. All arrows (yellow, red, green, pink, blue) indicate separated signals of 2p23 proximal and distal probes. The results shows a break-apart fluorescence *in situ* hybridization assay of the tumor cells with rearrangement of the gene encoding ALK. ALK, anaplastic lymphoma kinase.

The patient was treated with first-line chemotherapy consisting of cisplatin (75 mg/m^2^) and pemetrexed (PEM) (500 mg/m^2^) every three weeks, while simultaneous mutation analysis of the epidermal growth factor receptor (EGFR) gene was performed. However, no marked response was observed following two cycles of treatment. The subsequent EGFR mutation analysis revealed a L858R point mutation of exon 21 and a Q787Q point mutation of exon 20. Therefore, erlotinib was administered orally at a dose of 150 mg daily as the second-line therapy. A partial response was obtained one month after this targeted therapy and the prolonged overall tumor shrinkage lasted 25 months until the patient went to Cuba for CIMAvax-EGF vaccine therapy. CIMAvax EGF is a therapeutic anticancer vaccine developed entirely in Cuba and licensed in Cuba for use in adult patients with stage IIIB/IV NSCLC (10). The vaccine was administered at 4 anatomical sites (in 2 deltoid and 2 gluteus muscles), distributed at a dose equivalent to 2.4 mg of the antigen, corresponding to 0.6 mg of EGF in 1.2 ml water in oil emulsion per site on days 1, 7, 14 and 28, and monthly afterwards. The disease was stable for 6 months until a chest CT scan showed an enlarged primary lesion and increased pulmonary metastases. A combination of erlotinib (150 mg/day) treatment and cisplatin (75 mg/m^2^) + pemetrexed (500 mg/m^2^) chemotherapy were suggested subsequently. Subsequent to the completion of three cycles of treatment, no evident changes were documented on the chest CT, but a grade 4 (according to the Common Terminology Criteria for Adverse Events version 4.0) increase in alanine aminotransferase (ALT) and aspartate aminotransferase (AST) developed (11). The erlotinib treatment and chemotherapy were stopped, and the hepatoprotective agents, magnesium isoglycyrrhizinate, polyene phosphatidylcholine and reduced glutathione, were administered to the patient. A repeat biopsy was also conducted. DNA sequencing and fluorescence *in situ* hybridization analysis were used to detect the presence of EGFR and KRAS mutations and the ALK gene, respectively. The result revealed the wild-type KRAS and EGFR genes and ALK rearrangements (Fig. 3). Considering the shift in mutation status of the EGFR gene and the ALK rearrangements, the patient was treated with crizotinib (250 mg, twice a day) when the AST and ALT levels returned to normal. Slow progress was observed after 30 days, but the patient maintained a stable disease (SD) state according to the response evaluation criteria in solid tumors (12). Crizotinib treatment was continued until the last follow-up. Written informed consent was obtained from the patient for publication of this case study and the accompanying images.

## Discussion

EGFR-TKIs demonstrate efficacy in the treatment of patients with NSCLC who harbor activating EGFR mutations. These patients develop disease progression following a median response time of 10–14 months (13). The acquired resistance is unavoidable due to a number of different mechanisms, including c-Met amplification, activation of alternative pathways, T790M and tumor heterogeneity (14). The inconsistent status of EGFR mutations, also called heterogeneity, is believed to be associated with the secondary mutation of tumor cells, or to have existed during the transformation of a normal cell to a cancerous cell. A study by Shimizu *et al* (15) demonstrated the difference on the distribution of EGFR mutations between primary tumors and metastatic lymph nodes (MLNs) in patients with resected NSCLC, and indicated that the EGFR mutation status of MLN is a predictive marker of the response to EGFR-TKI therapy. Bai *et al* (16) provided evidence that chemotherapy may affect EGFR mutation status in tissue and peripheral blood samples. It was also reported that genetic changes associated with crizotinib resistance are heterogeneous in NSCLC patients with ALK rearrangements who respond to crizotinib and subsequently develop resistance (17). Therefore, secondary biopsies of growing tumors at the onset of clinical progression are crucial for guiding the subsequent treatment, although this is often not easy in clinical practice (18).

In the present case, the patient benefited from targeted therapy for more than two years and the EGFR mutation status changed following a series of treatments and disease progression. However, confirmation is required to assess whether the shift was derived from the chemotherapy, immunotherapy or the ALK rearrangements. The patient showed a good response to the erlotinib treatment initially, but not to the crizotinib. This indicates an association between the secondary mutations in kinases and the drug resistance to TKIs. The case should also highlight the fact that repeat biopsies for genomic evolution are necessary to aid in the clarification of the mechanism behind the development of the acquired drug resistance to TKIs, and that this may pave the way for the selection of appropriate treatments (19).
